# Asymmetric negative transfer effects of working memory training

**DOI:** 10.3758/s13421-023-01412-8

**Published:** 2023-04-21

**Authors:** Nan Ni, Susan E. Gathercole, Dennis Norris, Satoru Saito

**Affiliations:** 1 Graduate School of Education, Kyoto University, Yoshida-honmachi, Sakyo-ku, Kyoto, 606-8501 Japan; 2 Department of Psychiatry, University of Cambridge, Cambridge, UK; 3 MRC Cognition and Brain Sciences Unit, University of Cambridge, Cambridge, UK

**Keywords:** Working memory training, Cognitive routine, Transfer effect

## Abstract

Gathercole et al. (*Journal of Memory and Language*, *105*, 19–42, 2019) presented a cognitive routine framework for explaining the underlying mechanisms of working memory (WM) training and transfer. This framework conceptualizes training-induced changes as the acquisition of novel cognitive routines similar to learning a new skill. We further infer that WM training might not always generate positive outcomes because previously acquired routines may affect subsequent task performance in various ways. Thus, the present study aimed to demonstrate the negative effects of WM training via two experiments. We conducted Experiment 1 online using a two-phase training paradigm with only three training sessions per phase and replicated the key findings of Gathercole and Norris (in prep.) that training on a backward circle span task (a spatial task) transferred negatively to subsequent training on a backward letter span task (a verbal task). We conducted Experiment 2 using a reversed task order design corresponding to Experiment 1. The results indicated that the transfer from backward letter training to backward circle training was not negative, but rather weakly positive, suggesting that the direction of the negative transfer effect is asymmetric. The present study therefore found that a negative transfer effect can indeed occur under certain WM training designs. The presence of this asymmetric effect indicates that backward circle and backward letter tasks require different optimal routines and that the locus of negative transfer might be the acquisition process of such optimal routines. Hence, the routines already established for backward circle might hinder the development of optimal routines for backward letter, but not vice versa.

**Supplementary Information:**

The online version contains supplementary material available at 10.3758/s13421-023-01412-8.

## Introduction

Working memory (WM) refers to the ability to hold information in mind and mentally manipulate it over short periods in the face of distraction (Allen et al., 2009; Baddeley, 1992; Baddeley & Hitch, 1974; Cowan, 2008). WM is strongly correlated with not only higher-level cognitive functions including fluid intelligence (Engle et al., 1999; Kane et al., 2005; Unsworth et al., 2014) and cognitive control (Kane et al., 2001; Kane & Engle, 2003), but also cognitive performance in everyday life, such as reading comprehension, mental arithmetic, reasoning, and academic achievements (Cowan et al., 2005; Gathercole et al., 2008; Kane et al., 2007; Otsuka & Osaka, 2015; Tsubomi & Watanabe, 2017). Therefore, it is not surprising that researchers have attempted to improve individuals’ WM function through WM training, of which positive transfer effects are now known to be divided into near and far transfer. Near transfer is enhanced performance in a task intended to measure the trained cognitive domain (i.e., WM), while far transfer is the improvement of cognitive tasks in other domains such as fluid intelligence (Melby-Lervåg et al., 2016; Rowe et al., 2019; Soveri et al., 2017). Jaeggi et al. (2008) reported improved fluid intelligence in young adults by training their WM through computerized adaptive practice in a dual N-back task.

However, recent research following the first surge of the literature nearly two decades ago (Jaeggi et al., 2008; Klingberg et al., 2002) has largely failed to demonstrate far transfer, highlighting the methodological shortcomings of initial studies (e.g., Chooi & Thompson, 2012; Harrison et al., 2013; Redick et al., 2013; Thompson et al., 2013). Indeed, recent meta-analyses and reviews have indicated that WM training induces only a narrow range of outcomes. That is, while the performance on many WM tasks can be improved by training, the benefits of the training rarely transfer to other activities that also depend on WM such as intelligence tests or measures of real-world cognitive skills like reading comprehension and mental arithmetic (Melby-Lervåg et al., 2016; Melby-Lervåg & Hulme, 2013; Redick, 2019; Rodas & Greene, 2022; Rowe et al., 2019; Sala & Gobet, 2017, 2019; Smid et al., 2020; Soveri et al., 2017; Tsubomi et al., 2019; Watrin et al., 2022). Studies examining training outcomes within the WM domain further suggest that near transfer is also limited to novel tasks that share common features with trained tasks (Gathercole et al., 2019; Soveri et al., 2017).

Moreover, the WM training literature is often criticized for the lack of theory-based approaches explaining the mechanism underlying WM training and transfer (Redick, 2019; Smid et al., 2020). Previous theories of WM training fail to either explain the limited training outcomes or predict the conditions under which far and near transfer should or should not occur (for a review, see Gathercole et al., 2019).

To provide a more systematic mechanism for WM training, Gathercole et al. (2019) presented a cognitive routine framework that conceptualizes training-induced changes in WM as the acquisition of novel cognitive routines. As most WM training studies involve participants performing complex WM tasks that cannot be supported by existing mechanisms, they must coordinate and execute existing processes in a novel sequence to meet unfamiliar cognitive requirements. These cognitive procedures (i.e., cognitive routines) become more efficient and automatic with training, leading to improved performance on trained tasks. This development and sophistication of the new cognitive routine are similar to learning any new cognitive skill (Taatgen, 2013), and primarily depend on problem-solving abilities rather than expanding existing WM capacities. The (positive) transfer arises only when a new routine can be successfully adapted to an untrained task that shares task requirements in common. In addition, training on tasks supported by existing mechanisms does not require new routines, and hence generates weak and narrow transfer (Gathercole et al., 2019; Norris et al., 2019a). A more recent view of strategy development during WM training is also consistent with the cognitive routine framework’s notion of WM training as cognitive skill learning. This strategy mediation account captures training outcomes as the improved efficacy of unchanged WM capacity (Dunning & Holmes, 2014; Fellman et al., 2020; Forsberg et al., 2020; Laine et al., 2018; Malinovitch et al., 2021). Gains in trained tasks thus reflect the development of efficient task-specific strategies, which then give rise to near transfer when strategies can be applied to structurally similar untrained tasks (Fellman et al., 2020).

Although the cognitive routine framework requires further examination, we can infer from this theory that participants acquire new cognitive routines during training and that the routines generated previously may affect subsequent task performance. This assumption provides a new perspective for systematically assessing the WM training literature. We further consider the variability in routines and transfer effects this could bring about. There are two main types of routine variability. The first is the variability of routines across individuals: individuals may generate different routines when training on the same WM task. That is, they may tackle the same complex cognitive task differently, partly because their initial cognitive abilities can vary. For instance, individuals reporting vivid or poor mental imagery activate their brain networks differently in a mental rotation task, indicating that participants perform the same task in different ways (R. Logie, 2018; R. H. Logie et al., 2011). This routine variability in the same training could lead to different transfer effects among individuals. The second type is the variability of routines across tasks: training on different tasks results in the generation of different routines. In other words, participants may find a common solution when facing one task and another solution when facing another task. These various routines may affect the following task performance differently. This idea is supported by the cognitive routine framework as well as studies demonstrating limited cross-paradigm transfers within WM (Holmes et al., 2019; Soveri et al., 2017).

The above inferences from the cognitive routine framework suggest that the well-examined and hotly debated transfer effect in the WM training literature captures only half of the story. Previous WM training research normally assumes that although far transfer is absent and near transfer is limited, training will not generate a negative outcome. However, accepting the idea that different routines affect subsequent task performance differently, we could naturally reason that the transfer effect may lie on a continuum ranging from positive to null to negative rather than be categorized using a binary classification. Therefore, the transfer effects identified in previous studies should be classified as positive training outcomes.

We therefore infer the existence of potential negative training effects from the perspective of cognitive routine theory. Intensive training on a single cognitive task in the long term enhances the performance of the trained task and generates a positive transfer when the cognitive routines acquired in the previous training can be applied to the untrained task. However, under certain circumstances, these routines may constrain or even mislead trainees, preventing them from performing subsequent novel tasks optimally. More specifically, the cognitive routine developed in the previous training could be incompatible with the following task requirements and hinder the acquisition process of the new optimal routine for the novel task. Furthermore, it remains unknown whether repeated training on a single WM task produces something similar to “functional fixation” and lowers individuals’ cognitive flexibility and intelligence.

In summary, together with previous empirical evidence and the cognitive routine framework, we suppose that the present WM training paradigm only leads to a restricted pattern of positive transfer and might even produce negative outcomes. Thus, the primary goal of this study is to demonstrate the negative effects of WM training.

Gathercole and Norris (in prep.) developed a new two-phase training paradigm and, unexpectedly, provided the first evidence of a negative transfer from the first to the second training phase. Their two-phase training paradigm was proposed to address whether the transfer of WM training can be promoted through re-training, as common pre- and post-transfer tests may provide insufficient time to adapt trained routines to new tasks. In their initial study, the participants received 15 sessions (i.e., 15 days) of training on a certain WM task in Phase 1 and then 15 sessions of training on another WM task in Phase 2. The training results showed that those participants who first received training on a backward circle span task (a spatial task) performed worse in subsequent training on a backward letter span task (a verbal task) than the active control group, indicating that Phase 1 training negatively transferred to Phase 2 training. The “transfer” mentioned here and in the present study refers to the transfer from the previous training to the subsequent re-training, which is different from the gain detected by the “one-shot” post-transfer tests of previous WM training studies. The lower performance of backward letter span training may reflect the disruptions caused by incompatible cognitive routines (Norris et al., 2019a). This finding is consistent with the prediction of training-induced negative effects derived from the cognitive routine framework.

To provide further evidence for this negative transfer effect, we conducted two experiments in this study. Table 1 summarizes the training designs of Experiments 1 and 2. Experiment 1 aimed to partially replicate the finding of the effect demonstrated by Gathercole and Norris (in prep.), but with only three as opposed to 15 training sessions in each phase. The rationale for this adaption is that in the original and similar studies, training performance grew rapidly in the first three training sessions of each phase and then only gradually improved as training proceeded (Gathercole & Norris, in prep.; Norris et al., 2019a). We thus speculated that participants established the primary routines in the initial stage and only continued to refine them in the later training. In addition, research on strategy development has indicated that participants normally generate task-specific strategies during the early stages of training (Fellman et al., 2020; Forsberg et al., 2020; Laine et al., 2018). Hence, we might still find a reliable negative transfer effect in this shorter training design.

**Table 1:** The training designs of Experiment 1 and Experiment 2

	Group no.	Phase 1 training task	Phase 2 training task
Experiment 1	1	Backward digit span	Backward letter span
	2	Backward circle span	Backward letter span
	3 (active control)	Color change detection	Backward letter span
Experiment 2	1	Backward square span	Backward circle span
	2	Backward letter span	Backward circle span
	3 (active control)	Color change detection	Backward circle span

Experiment 1 consisted of two adaptive training phases. In Phase 1, the participants were divided into three groups trained on a (1) backward digit span task, (2) backward circle span task, and (3) color change detection task (active control) for three sessions. In Phase 2, all the participants were trained on a backward letter span task for three sessions. The training tasks were adapted from Norris et al. (2019a). The backward digit span and backward letter span tasks are verbal backward serial recall tasks extensively used to measure intelligence and other complex cognitive abilities (Norris et al., 2019b). They require participants to recall a sequence of verbal stimuli in the reverse order of the presentation, and backward recall is usually slower and less accurate than forward recall (Anders & Lillyquist, 1971; Donolato et al., 2017; Isaacs & Vargha-Khadem, 1989). The backward circle span, a variant of the forward circle span task used in previous research, requires the backward recall of spatial locations presented in a sequence (e.g., Minear et al., 2016; Norris et al., 2019a). In contrast to verbal serial recall, few performance differences between the forward and backward versions of spatial serial recall have been reported (Donolato et al., 2017). The color change detection task was developed by Luck and Vogel (1997) to measure the visual short-term memory capacity. Participants are required to detect changes in the colors of squares presented briefly. As participants do not need to maintain any serial order of the stimuli, this task serves as the ideal active control condition for the other two backward serial recall training groups (Norris et al., 2019a). According to the cognitive taxonomy of WM tasks in Gathercole et al. (2019), all these training tasks are considered to be unfamiliar and highly challenging tasks that require new routines. Therefore, we expected that performance on these tasks would improve significantly with training, thereby potentially generating transfer effects. Moreover, participants’ strategies for performing the backward serial recall of verbal and spatial stimuli have been systematically examined (Norris et al., 2019b), which provides a foundation for interpreting the training and transfer results of the present study.

We predicted that in Experiment 1, for the three training groups in Phase 1, participants’ performance would improve as the training proceeded, indicating the effectiveness of the training in Phase 1. In Phase 2, we predicted that the backward digit span training in Phase 1 would positively transfer to the backward letter span training, while the backward circle span training in Phase 1 would negatively transfer to the backward letter span training, replicating the results of Gathercole and Norris (in prep.). That is, in Phase 2, the backward digit span training group would outperform the active control condition on average, whereas the backward circle span training group would perform worse. We inferred that the lower performance of the backward letter span training may reflect the disruptions caused by the incompatible cognitive routines generated in the previous training phase.

Using a reverse task order design corresponding to Experiment 1, we conducted Experiment 2 to further verify the hypotheses from Experiment 1. As before, Experiment 2 involved two adaptive training phases (see Table 1). The three groups were first trained on a (1) backward square span task, (2) backward letter span task, or (3) color change detection task (active control) for three sessions in Phase 1. Then, all the participants were trained on a backward circle span task for three sessions in Phase 2. The critical manipulation was to reverse the order of the tasks in the negative transfer condition in Experiment 1 to explore whether a negative transfer effect would still occur. The backward square span task, a variant of the backward circle span task, simply replaces circular stimuli with similarly sized squares. This created the ideal condition to examine the possible positive transfer within the spatial domain, corresponding to the positive transfer within the verbal domain in Experiment 1.

For Experiment 2, we predicted that the Phase 1 performance of each group would also show a significant gain across the training. In Phase 2, with the backward circle span training, our prediction was that the participants of the backward square span training group would outperform the active control group, indicating a positive transfer from the previous training. However, owing to a lack of previous evidence, we did not hypothesize the Phase 2 performance of the backward letter span training group. Experiment 1 suggested that the cognitive routines generated in the backward circle span training may be incompatible with those in the backward letter span training. Therefore, one prediction was that in the reverse task order condition, Phase 1 training would also negatively transfer to Phase 2 training, as indicated by lower Phase 2 performance than for the other two groups. An alternative prediction was that the negative transfer induced by routine incompatibility is asymmetric as opposed to bidirectional. Although training on the backward letter span task is disrupted by the routines developed previously in the backward circle span training, those developed in the previous backward letter span training are not necessarily detrimental to the following training on the backward circle span task. If this is the case, the backward letter span training group would show similar training gains to those of the active control group. The hypotheses, methods, analysis plans, and data exclusion criteria of Experiment 2 were pre-registered using the Open Science Framework (https://osf.io/9ethj).

## Experiment 1

### Method

#### Participants

Sixty undergraduate and graduate students were recruited through advertisements at Kyoto University. Our participants received 4,000 Japanese yen to participate. The inclusion criteria were as follows: at least 18 years old, native Japanese speaker, normal eyesight and hearing ability (sufficient for performing experimental tasks on computers), and no current psychiatric or neurological illnesses. The participants were randomly allocated to the three training conditions in Phase 1; thus, there were 20 participants in each condition. This sample size per group was based on the meta-analysis results of the near transfer effect following WM training by Gathercole et al. (2019) and among the standard range of many previous WM training studies (e.g., Dunning & Holmes, 2014; Norris et al., 2019a). We used the software program G*Power 3.1 (Faul et al., 2007, 2009) to conduct a power analysis. To compare the between-participant factor (training condition) in a repeated-measures analysis of variance (ANOVA) model, this sample size delivered a power of .92 to detect a large effect size, f = .40. However, after excluding data that did not meet certain criteria, a final sample of 45 participants was included in the analyses (see the details and rationales of the exclusion criteria in the *Analysis plan* subsections). This sample size still yielded an acceptable power of .82 to detect a large effect of the training condition. Table 2 summarizes the demographic characteristics of the final sample.

**Table 2:** Demographics and baseline analyses of Experiment 1

	Digit	Circle	Color	*p*	BF_10_
*N*	15	14	16	-	-
Age, y	22.5 (4.67)	21.6 (2.79)	21.1 (2.22)	-	-
Gender F/M	6/9	6/8	6/10	-	-
Forward letter span	5.07 (0.88)	4.79 (0.98)	5.38 (1.03)	0.258	0.435
Forward letter score	218.33 (17.20)	210.93 (27.25)	224.44 (25.85)	0.311	0.379
Backward letter span	4.93 (1.58)	4.86 (1.23)	5.06 (1.24)	0.916	0.175
Backward letter score	223.60 (29.24)	210.79 (31.31)	222.94 (36.15)	0.496	0.269

*Note*: Values in parentheses are standard deviations

#### Procedure

All the tasks were performed online on the participants’ personal computers. On the first day of the experiment, the experimenter provided oral instructions through the Zoom video conference software. The participants then completed the two baseline tasks, namely, the forward and backward letter span tasks. In Phase 1, each participant completed the (1) backward digit span training, (2) backward circle span training, or (3) color change detection training for three sessions. In Phase 2, all the participants completed the backward letter span training for three sessions. They were instructed to complete one training session daily, between 7 a.m. and 11 p.m., on a Google Chrome browser. The estimated session time was 30–40 min. After the final training session, the participants completed a strategy use questionnaire.

#### Material

##### Training tasks

The training tasks were adapted from the tasks in Norris et al. (2019a). The programming of the tasks used a JavaScript library to run behavioral experiments in web browsers, jsPsych ver.6.1.0 (de Leeuw, 2015), and referred to the tasks in Experiment Factory ver. 3.1.0 (Sochat, 2018).

*Backward digit span task.* In one trial, the fixation mark “+” was first presented in the middle of the screen for 750 ms. Then, a sequence of digits was displayed at a rate of 500 ms per digit, with a blank interval of 250 ms between digits. At the end of the sequence, a numeric panel (digits 1–9 in a 3 × 3 telephone keyboard layout) was displayed, and the participants were required to click the buttons in the reverse order of the sequence. When the sequence length was nine or less, digits were randomly drawn from 1 to 9 without replacement. When the array length was longer than nine, the first nine digits were randomly drawn from 1 to 9 without replacement and this process was repeated from the tenth item. No digits appeared successively twice and there were no three or more successive ascending or descending digits. There were eight blocks of ten trials in each training session. The number of digits presented (the span) varied adaptively. Training began with sequences of three digits; they increased by one when the participants answered eight or more trials correctly in a block and decreased by one if the participants answered two or fewer trials correctly. The beginning span of the next training session continued using the span reached in the last block of the previous session and could increase or decrease by one depending on the performance of the last block. The principal score for the analysis was the highest span reached in the eight blocks of each session.

*Backward circle span task.* In one trial, an array of pseudo-randomly positioned circles were presented. Each circle had a radius of 81 pixels and a minimum center-to-center separation of 272 pixels. (However, these circle settings were changed to a radius of 60 pixels and a minimum separation of 202 pixels for four participants, as their computer screen resolutions (e.g., 1,366 × 768, 1,400 × 900) were insufficient to display the nine circles simultaneously.) All the circles were colored light blue on a gray background and each circle turned dark blue for 250 ms in a random sequence. There was a 500-ms interval between each presentation. After the display of the sequence, all the circles remained visible in light blue, and the participants were required to click the circles in the reversed order of the displayed sequence. There were eight blocks of ten trials in each training session. Training began with sequences of three circles and the number of circles presented (the span) was varied adaptively according to the same criteria as in the backward digit span training. The principal score for the analysis was the highest span reached in the eight blocks of each session.

*Color change detection task.* In one trial, a display containing several colored squares was first presented for 250 ms. The squares of 38 pixels were randomly placed on the screen and the colors of the squares were chosen randomly with replacements from a set of seven identifiable colors. After a 1,000 ms blank retention interval, a probe was displayed for 500 ms. The probe repeated the previous square display, with the exception that one square was randomly chosen and surrounded by a larger red square. The color of this probed square either remained the same or was randomly changed to another color at an equal probability. The participants were required to judge whether the color of the probe square had changed by clicking the “same” or “changed” button at the bottom within 5,000 ms. Each training session consisted of eight blocks of 30 trials. The number of squares presented (span/set size) varied adaptively. Training began with three squares; these increased by one when the participants answered 27 or more trials correctly in a block and decreased by one if the participants answered 18 or fewer trials correctly. Cowan’s *K* was also computed for every block to measure performance, where capacity measure *K* = the set size of the block × (proportion hits – proportion false alarms) (Cowan, 2001; Cowan et al., 2005). The principal scores for the analysis were the highest span and highest capacity measure *K* reached in the eight blocks of each session.

*Backward letter span task.* The procedure of the backward letter span task was identical to that of the backward digit span task, with the exception that the stimuli were changed to nine consonants (B, F, H, J, L, M, Q, R, and S). The letters on the recall panel were arranged in alphabetical order. Each training session had eight blocks of ten trials. The number of letters presented (the span) was varied adaptively using the same criteria as in the backward digit span training. The principal score for the analysis was the highest span reached in the eight blocks of each session.

##### Baseline tasks

*Baseline forward letter span task.* The same presentation procedure was employed as in the backward letter span training. The participants were required to click on the buttons in the exact order in which the items appeared. The baseline test began with a block of six trials with a sequence of three letters and increased by one in the next block until the sequence length reached ten. The span of the baseline was determined as the longest sequence length for which four or more sequences were correctly recalled. None of the participants reached span 10 in the baseline task. The recall score of each trial was also calculated using the scoring method of McKelvie (1987), which was originally designed to measure Hebb recall performance. This method accounts for both position and serial order to provide a more subtle measure of recall performance in each trial (McKelvie, 1987; Smalle et al., 2016). The overall performance score was the summation of the recall score in all 48 trials. The scores for the analysis were the span reached and performance score.

*Baseline backward letter span task.* The procedure of this backward task was identical to that of the forward task, with the exception that the participants were instructed to answer in the reverse order of the sequence. The span reached and performance score were used in the analysis.

##### Strategy use questionnaire

This questionnaire was based on Gathercole and Norris (in prep.) and translated into Japanese. After the final training session, the participants completed a series of questions about their strategy use during Phases 1 and 2. Several specific strategies were provided, and the participants answered by selecting the frequency on a scale of 0–3, with 0 being “never,” 1 being “occasionally,” 2 being “frequently,” and 3 being “almost always.” The questions are summarized in the Online Supplementary Material (OSM) section D. In particular, we added a new question described as “use the panel as recall cues” into the Phase 1 questionnaire for the backward digit training group and the Phase 2 questionnaire for all the groups because we speculated that this strategy could be easily induced by backward circle training and hindered the following backward letter training. The participants were also encouraged to describe the strategy they used in as much detail as possible if they used strategies not mentioned in the questionnaire.

#### Analysis plan

We used the standard *p* < .05 criterion to determine whether one-way ANOVAs, repeated-measures ANOVAs, mixed ANOVAs, and the post hoc test using the Holm correction suggested that the results were significantly different from those expected if the null hypothesis was supported. We also reported Bayes factors (BFs) when needed to examine the null effect of the baseline differences or training conditions. Frequentist statistics analyses and Bayesian analyses were conducted using JASP ver. 0.16.3 (JASP Team, 2022; Love et al., 2019; Wagenmakers et al., 2018).

Before the analyses, the data were screened according to the following exclusion criteria. As Experiment 1 was conducted online, the data quality was inevitably lower than that usually acquired in a standard laboratory environment. Some of the participants failed to follow the instructions and some lost concentration during training, while the data file was not correctly saved to the server because of Internet connection problems on other occasions. Therefore, we had to carefully develop exclusion criteria to rule out questionable data. The details and rationale of the exclusion criteria are described below. To ensure transparency, the raw dataset before and after exclusion is available via the Open Science Framework (https://osf.io/gmybk/?view_only=7b30719ef8204cedbfd359de81aedb16).

In the baseline testing as well as the Phase 1 and Phase 2 training, if one of the following situations existed, the participant’s data were excluded from the analysis. The first criterion was (1) lost data or (2) task incompletion because of technical failure or the participants’ failure to follow the instructions. The second criterion was that the span in all the training blocks dropped to 2 or had a successive drop of three levels. This indicated that the participants had not concentrated during the training. In Phase 2, if the following situation existed, the participant’s data were excluded from the analysis. The third criterion was that the average span of 16 blocks in the session 2 and session 3 training in Phase 2 was lower than the span reached in the baseline backward letter span task. This indicated that the participants had not concentrated during the training, as their performance worsened on average compared with their baseline.

For the spans and performance scores in the two baseline tasks, we used one-way ANOVAs to analyze the group differences. The manipulated independent variable was the training task in Phase 1 and the dependent variable was the span or performance score of the baseline tasks. We report the results of both the traditional null-hypothesis significance testing (NHST) ANOVAs and the Bayesian ANOVAs.

The Phase 1 training outcomes for the three groups were separately analyzed using repeated-measures ANOVAs, as the training tasks between the groups differed. The repeated-measures factor was the training sessions in Phase 1 and the dependent variable was the highest span or highest capacity measure *K* reached in each training session in Phase 1.

The Phase 2 training outcomes were analyzed in a mixed ANOVA with the training task in Phase 1 as the between-participant factor and the training day in Phase 2 as the repeated-measures factor. The dependent variable was the highest span reached in each training session in Phase 2. The span of baseline backward letter span task was included as a covariate. In an additional exploratory analysis, we fitted the overall Phase 2 training results into a linear mixed-effects model to capture the performance changes across the 24 training blocks of the three training sessions.

To evaluate the strategy usage of Phase 2 training, we also performed the NHST and Bayesian one-way ANOVAs to analyze whether there was a group difference in strategy use.

### Results

#### Exclusions and baseline data

According to the first, second, and third criteria, six, six, and three participants, respectively, were excluded from the following analyses. For the four scores in the two baseline tasks, the NHST results indicated no significant differences between the groups at baseline (all *p*s > .05). Compared with the null model, although the Bayesian outcomes of the baseline forward letter span task were equivocal, providing weak evidence towards the null hypothesis, the outcomes of the baseline backward letter span task supported the null hypothesis. Table 2 summarizes the analyses of the baseline tasks.

#### Training data

**Fig. 1:**
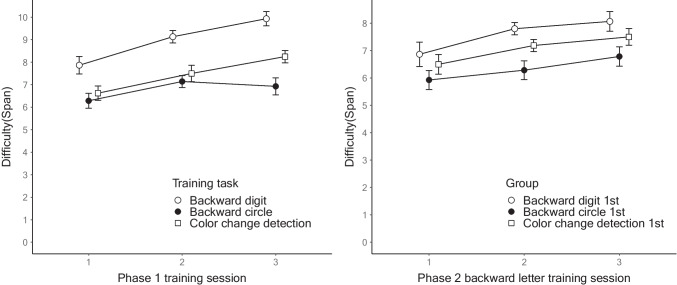
Performance changes in Experiment 1. *Note*: The left panel shows the performance changes of each training group in Phase 1. The right panel shows the performance changes of the backward letter training in Phase 2. The error bars represent 95% within-participant confidence intervals

Figure 1 shows the highest scores for each training session in the two phases. For each training group in Phase 1, the repeated-measures ANOVAs indicated significant increases in performance across the training: backward digit, *F*(2, 28) = 45.207, MSE = 0.360, *p* < .001, η_p_^2^ = 0.764; backward circle, *F*(2, 26) = 8.593, MSE = 0.324, *p* < .01, η_p_^2^ = 0.398; color change detection span, *F*(2, 30) = 29.308, MSE = 0.361, *p* < .001, η_p_^2^ = 0.661; and color change detection capacity *K*, *F*(2, 30) = 4.401, MSE = 0.518, *p* < .05, η_p_^2^ = 0.227.

**Fig. 2:**
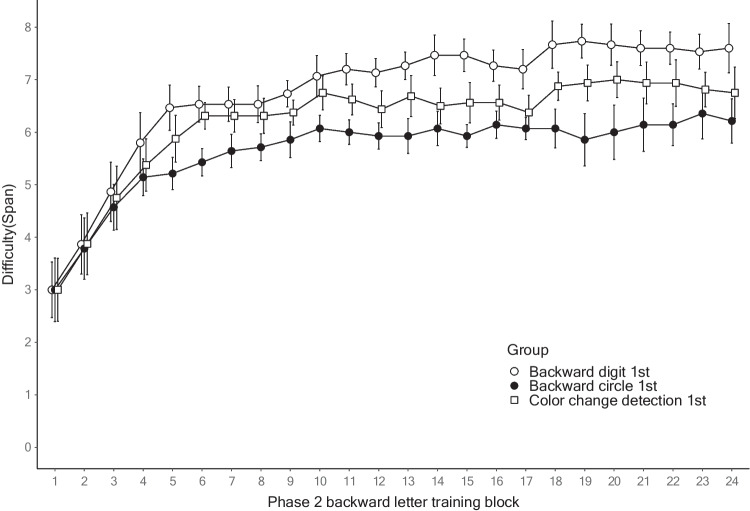
Performance of every block in Phase 2 of Experiment 1. *Note*: This figure plots the span of Phase 2 training on all the training blocks as a function of the training group in Phase 1*.* The error bars represent 95% within-participant confidence intervals

For Phase 2, a 3 (group) × 3 (training session) mixed ANOVA was performed on the highest scores of each backward letter training session, with the baseline backward letter span as the covariate. There was a significant main effect of the Phase 1 training group, *F*(2, 41) = 12.284, MSE = 1.249, *p* < .001, η_p_^2^ = 0.375. The post hoc analyses of average performance using the Holm correction showed that the digit group had significantly higher scores than the color and circle groups, and the circle group had significantly lower scores than the color group (active control). In addition, we observed a significant main effect of the covariate, *F*(1, 41) = 102.405, MSE = 1.249, *p* < .001, η_p_^2^ = 0.714, and a significant interaction between the training session and baseline backward letter span, *F*(2, 82) = 3.763, MSE = 0.341, *p* < .05, η_p_^2^ = 0.084. The non-significant main effect of the training session, *F*(2, 82) = 0.194, MSE = 0.341, *p* = 0.824, η_p_^2^ = 0.005, may have resulted from the significant interaction between the training session and covariate. We conducted an additional analysis to examine the correlation between the baseline backward letter span and training gain from sessions 1 to 3, finding a moderate positive correlation, *r*(43) = .329, *p* < .05. In line with previous WM training research (e.g., Foster et al., 2017), this result indicates that the participants who performed better at baseline seemed to gain more from the training. The group × training session interaction was not significant (*F*(4, 82) = 0.872, MSE = 0.341, *p* = 0.485, η_p_^2^ = 0.041). The exploratory linear mixed-effects analysis examining the block-level improvement in Phase 2 replicated the main findings in the mixed ANOVA (see OSM B). Figure 2 plots the span of Phase 2 backward letter training on the 24 training blocks as a function of the training group in Phase 1.

#### Strategy questionnaire results

For the NHST ANOVAs, there were no significant group differences in any of the strategy statements (all *p*s > .05). The Bayesian outcomes also indicated evidence for the null hypothesis in each statement. The results are reported in OSM E.

### Discussion

As the performance of all three groups improved across the Phase 1 training, we concluded that it was effective even though the training duration was shortened to three sessions. The top half of Table 3 summarizes the performance gain of the span in Experiment 1. It shows the average performance gain from the first session to the third session in both Phase 1 and Phase 2 as well as the average gain from the baseline (backward letter span task for Experiment 1 and backward circle span task for Experiment 2 to the third session of Phase 2. The gains were overall smaller than those in a previous study of 20 training sessions (Norris et al., 2019a). Further, the data on the backward circle training indicated a more limited period of training-related gains, with little gain from sessions 2 to 3. Hence, this group yielded smaller training-related gains than the other two groups.

**Table 3:** Performance gain of the span in Experiment 1 and Experiment 2

Experiment no.	Phase 1		Phase 2		
	Training task	Sessions 1–3	Training task	Sessions 1–3	Backward baseline to Session 3
1	Digit	26.0%	Letter	17.0%	72.3%
	Circle	10.3%	Letter	15.3%	44.3%
	Color	24.8%	Letter	16.2%	52.5%
2	Square	8.8%	Circle	8.7%	45.1%
	Letter	23.7%	Circle	13.6%	39.3%
	Color	28.0%	Circle	10.0%	30.5%

*Note*: As for the capacity *K* in the color change detection training, the gain was 15.9% in Experiment 1 and 23.0% in Experiment 2

For Phase 2, the backward letter training gains were enhanced by the previous backward digit training relative to the color change detection training (the active control condition), indicating that the Phase 1 training transferred positively to the Phase 2 training. By contrast, the previous backward circle training led to diminished gains in the backward letter training compared with the active control group, indicating that the Phase 1 training transferred negatively to the Phase 2 training. The Phase 2 performance of the backward circle group was markedly lower from the beginning of the training. Thus, as demonstrated by Gathercole and Norris (in prep.), we replicated the pattern of the negative transfer effect across the two training phases. This decreased performance was also detected in our pilot experiment (*N* = 15), which was conducted in a mixed laboratory and online environment. The results of the pilot experiment yielded an even larger effect size for the training conditions, η_p_^2^ = 0.640 (see OSM A).

## Experiment 2

### Method

#### Participants

Sixty undergraduate and graduate Kyoto University students meeting the same criteria as in Experiment 1 were recruited and paid 4,000 Japanese yen to participate. They were randomly allocated to the three training conditions of Phase 1 (20 per condition). The results of Experiment 1 indicated a large effect size of the training conditions, η_p_^2^ = 0.375; therefore, we expected to detect a large effect size of the between-participant factor with the same sample size and a similar design. As Experiment 2 was also conducted online, we employed the same exclusion criteria as in Experiment 1, which proved to be valid. The final sample size of 47 participants still yielded a power of .84 to detect a large effect of the training conditions. Table 4 summarizes the demographic characteristics of the final sample.

**Table 4:** Demographics and baseline analyses of Experiment 2

	Square	Letter	Color	*p*	BF_10_
*N*	17	16	14	-	-
Age, y	20.7 (2.31)	21.0 (1.59)	21.7 (2.76)	-	-
Gender F/M	7/10	5/11	4/10	-	-
Forward circle span	6.82 (0.95)	6.75 (0.68)	6.93 (1.00)	0.859	0.178
Forward circle score	219.29 (14.28)	215.31 (14.06)	219.00 (13.63)	0.673	0.212
Backward circle span	5.94 (0.97)	5.75 (0.78)	5.79 (0.80)	0.794	0.189
Backward circle score	210.00 (11.00)	207.25 (10.54)	208.29 (12.48)	0.780	0.191

*Note*: Values in parentheses are standard deviations

#### Procedure

The training procedure was identical to that of Experiment 1, except that the training and baseline tasks differed. Before Phase 1, the participants completed two baseline tasks: the forward and backward circle span tasks. In Phase 1, each participant completed the (1) backward square span training, (2) backward letter span training, or (3) color change detection training for three sessions. In Phase 2, all the participants completed the same backward circle span training for three sessions. After the final training session, the participants completed the strategy use questionnaire.

#### Materials

##### Training tasks

The backward letter span, backward circle span, and color change detection tasks were identical to those in Experiment 1. The backward circle span task was adapted such that instead of using absolute pixels, the radius of the circle was adjusted to 5% of the screen height of the participant’s device and the minimum center-to-center separation was adjusted to 15% of the screen height. These ratios were the approximate settings used in the forward circle span task of Norris et al. (2019a). These manipulations guaranteed sufficient space to display the nine circles simultaneously regardless of the participant’s computer screen resolution. The backward square span task was adapted from the backward circle span task by changing the stimuli to a similarly sized square. The side length of the square was 4.5% and the minimum center-to-center separation was adjusted to 15% of the screen height. The principal scores for the analysis in each group were the highest span or highest capacity measure *K* reached in each session.

##### Baseline tasks

The baseline forward circle span and baseline backward circle span tasks used the same procedure as in the backward circle span training. The participants were required to click on the circles in the exact (reverse) order in the forward (backward) task. The baseline tests began with a block of six trials with a sequence of three circles, and increased by one in the next block until the sequence length reached nine. The span of each baseline task was the longest sequence length for which four or more sequences were correctly recalled. None of the participants reached span 9 in the baseline tasks. The recall score for each trial was also calculated using the same method as in Experiment 1. The scores for the analysis were the span reached and performance score.

##### Strategy use questionnaire

We used the same questionnaire as in Experiment 1 and only added the question “use the panel as recall cues” into the Phase 1 questionnaire for the backward letter training group.

#### Analysis plan

The data were screened according to the same exclusion criteria as in Experiment 1. The final data of the 47 participants were then analyzed. We performed the same analyses as in Experiment 1 to evaluate the baseline differences, training outcomes of Phases 1 and 2, and strategy use reports.

### Results

#### Exclusions and baseline data

According to the first, second, and third criteria, two, three, and eight participants, respectively, were excluded from the following analyses. For the baseline tasks, the NHST and Bayesian one-way ANOVAs confirmed the absence of group differences (all *p*s > .05, and all Bayes factors (BF_10_) < 0.33). Table 4 summarizes the analyses of the baseline tasks.

#### Training data

**Fig. 3:**
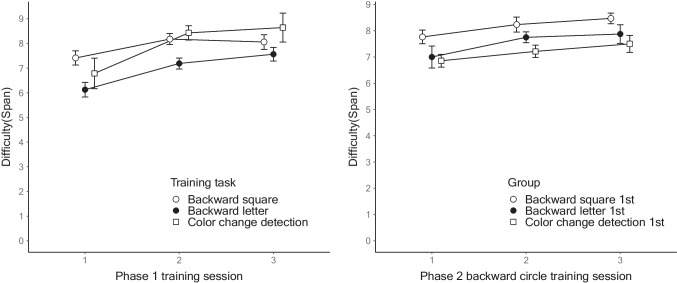
Performance changes in Experiment 2. *Note*: The left panel shows the performance changes of each training group in Phase 1. The right panel shows the performance changes of the backward circle training in Phase 2. The error bars represent 95% within-participant confidence intervals

Figure 3 shows the highest scores for each training session in the two phases. For Phase 1, the repeated-measures ANOVAs indicated significant performance gains across the training: backward square, *F*(2, 32) = 10.361, MSE = 0.278, *p* < .001, η_p_^2^ = 0.393; backward letter, *F*(2, 30) = 35.387, MSE = 0.251, *p* < .001, η_p_^2^ = 0.702; color change detection span, *F*(1.346, 17.492) = 17.813, MSE = 1.206, *p* < .001, η_p_^2^ = 0.578 (the Greenhouse–Geisser Correction was used to adjust for the lack of sphericity); and color change detection capacity *K*, *F*(2, 26) = 6.217, MSE = 0.829, *p* < .01, η_p_^2^ = 0.323.

**Fig. 4:**
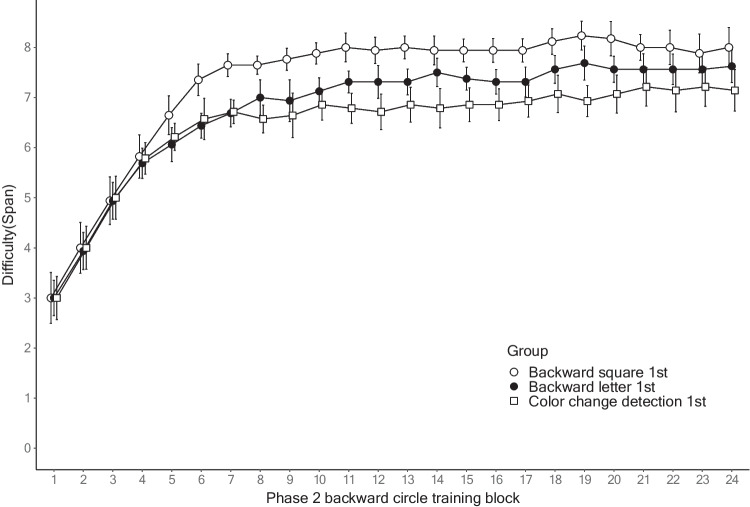
Performance of every block in Phase 2 of Experiment 2*. Note*: This figure plots the span of Phase 2 training on all the training blocks as a function of the training group in Phase 1*.* The error bars represent 95% within-participant confidence intervals

For Phase 2, a mixed ANOVA found the significant main effect of the Phase 1 training group, *F*(2, 43) = 4.206, MSE = 2.223, *p* < .05, η_p_^2^ = 0.164. The post hoc analyses of average spans using the Holm correction indicated that the square group had significantly higher spans than the color group (active control). The letter group performance was between that of the square and color groups (not significant). There was also a significant main effect of the covariate, *F*(1, 43) = 9.689, MSE = 2.223, *p* < .01, η_p_^2^ = 0.184. No other effects were significant including for the main effect of the training session, *F*(2, 86) = 0.495, MSE = 0.295, *p* = 0.611, η_p_^2^ = 0.011; the group × training session interaction, *F*(4, 86) = 0.554, MSE = 0.295, *p* = 0.697, η_p_^2^ = 0.025; and the training session × covariate interaction, *F*(2, 86) = 0.214, MSE = 0.295, *p* = 0.807, η_p_^2^ = 0.005. Again, the linear mixed-effects analysis of the spans across all the Phase 2 training blocks replicated the main findings in the mixed ANOVA (see OSM C). Figure 4 shows the span of the Phase 2 backward circle training on 24 blocks as a function of the training group in Phase 1.

#### Strategy questionnaire results

The NHST and Bayesian results indicated no group differences in strategy use, with the exception of strategies 1 and 3. For strategy 1 (“rehearse the items as they were presented”), although the Bayes factor was equivocal (BF_10_ = 1.773), there was a significant main effect of group, *F*(2, 44) = 3.533, MSE = 1.247, *p* < .05, η_p_^2^ = 0.138, with the letter group rating significantly higher than the color group. For strategy 3 (“group the items according to the pattern they form”), the main effect of the group was significant, *F*(2, 44) = 5.595, MSE = 0.935, *p* < .01, η_p_^2^ = 0.203, with the color group rating significantly lower than the other two groups. The Bayesian outcomes of strategy 3 also favored the alternative hypothesis (BF_10_ = 6.884). The results are reported in OSM F.

### Discussion

The bottom half of Table 3 summarizes the performance gain of the span in Experiment 2. Consistent with Experiment 1, the training was effective in Experiment 2. We also observed a smaller training-related gain in the backward training with spatial stimuli of squares and circles than in the training with verbal stimuli of letters.

For Phase 2, the results showed the benefit of earlier backward square training on the later backward circle training performance, indicating a positive cross-phase transfer effect. Note that both spatial tasks require participants to recall only the locations of the stimuli rather than their identities, unlike the digits and letters stimuli in Experiment 1. Therefore, it is not surprising to see a robust positive transfer, as Phase 2 involved essentially the same task as the preceding phase.

Critically, there was no sign of corresponding decreased performance in the backward circle training caused by the previous backward letter training. As shown in Fig. 4, the performance of the backward letter training group, although not significantly different from that of the other two groups, was slightly higher than that of the active control group. Thus, the transfer from the Phase 1 backward letter training to the Phase 2 backward circle training was mildly positive as opposed to negative.

## General discussion

This study investigated the negative transfer effect using a two-phase WM training design. In Experiment 1, we found a positive transfer from the backward digit training to the backward letter training and, more importantly, a negative transfer from the backward circle training to the backward letter training. Since this was initially demonstrated by Gathercole and Norris (in prep.) and then replicated in our pilot experiment and Experiment 1, we conclude that this negative cross-phase transfer has a relatively robust effect. In Experiment 2, the results indicated only a positive transfer from the backward square training to the backward circle training. The backward letter training followed by the backward circle training did not demonstrate any negative transfer. Thus, the present study showed that positive cross-phase transfer can occur within both the verbal and the spatial domains when tasks share similar structures. Moreover, the negative transfer effect was asymmetric. It was found only in Experiment 1, but not in Experiment 2, which had a reverse task order design.

The difference in the magnitude of the training-related gains between the backward circle and backward letter training may have partly contributed to the transfer asymmetry. Indeed, in both experiments, we observed that the overall Phase 1 performance gain in the backward training using spatial stimuli (10.3% for the circle, 8.8% for the square) was smaller than the gain in the training using verbal stimuli (23.7% for the letter, 26.0% for the digit). Especially for the backward circle training in Experiment 1, we found a more limited period of training-related gains only from sessions 1 to 2, which also led to the relatively small gains. Presumably, the greater the participants’ gains in Phase 1, the more likely those gains, which are supposed to be the improved performance in backward serial recall tasks, were taken into Phase 2. In Experiment 1, the backward digit training group benefited more from Phase 1 and thus demonstrated a positive transfer in the Phase 2 backward letter training. By contrast, the gains from the Phase 1 backward circle training might have been insufficient to produce a reliable transfer. This fact could explain the large performance difference between the backward circle group and the backward digit group in Phase 2. In the same vein, in Experiment 2, the smaller performance difference between the backward square group and the backward letter group in Phase 2 might have been due to the benefit from the larger gains obtained during the backward letter training in Phase 1.

However, the difference in gains alone cannot account for the negative transfer in Experiment 1, which is the lower performance of the backward circle group than the active control group. Since the control group received color change detection training, no gain should be associated with performing backward serial recall and thus no transfer should materialize. Therefore, any gain from the Phase 1 backward circle training should have been reflected in Phase 2 by a slightly positive transfer rather than a negative transfer. Furthermore, the gain itself also failed to explain the more robust positive transfer from the backward square training to the backward circle training in Experiment 2, since there were smaller gains in the Phase 1 backward square training. Another concern raised by the magnitude of the training-related gains is that the overall limited gains in the Phase 2 backward circle training might have made it difficult for the researchers to detect the negative transfer effect in Experiment 2. However, this is also unlikely because the Phase 2 performance of the backward letter training group was actually above that of the active control group at the descriptive level, thereby showing the opposite pattern of a mildly positive transfer.

Instead, the cognitive routine framework (Gathercole et al., 2019) may provide a plausible explanation for the current findings. According to this framework, during training on demanding cognitive tasks, participants develop new cognitive routines similar to learning a new skill. A (positive) transfer arises only when a routine can be applied to a novel task that shares common task features. We then infer that previously acquired routines could affect subsequent cognitive activities in various ways. Therefore, certain routines may lower the performance of the following task, as confirmed in this study. This finding provides vital evidence for the cognitive routine framework, as no previous accounts have predicted the existence of such a training-induced negative effect.

The poorer performance of the backward letter training following the backward circle training may reflect the disruptions caused by the incompatible cognitive routines generated in the previous training phase (Norris et al., 2019a). However, the designs of the present study do not allow us to specify which part of the routine is incompatible when applied to the subsequent training phase. At the least, this type of routine incompatibility is not reflected in our strategy use questionnaire. One explanation is that the incompatibility may not be at the level of task strategy, the higher-level cognitive routine structure. This indicates that as a complex of automated cognitive procedures, not all parts of the cognitive routine are verbalizable like strategy. In other words, some parts of the routine are not available to participants’ introspection.

Further, the transfer asymmetry suggests that the negative effect may not simply result from a conflict between the two cognitive routines. Taken together with the evidence from Experiment 1 that the Phase 2 performance of the backward circle group was markedly lower from the beginning of training, we infer that the locus of the disruptions might be in the acquisition process of optimal routines. Although the backward circle and backward letter span tasks are both serial order recall tasks, separated independent routines should be responsible for them as opposed to one general routine. The optimal routine for the first task is established in the Phase 1 training. The Phase 2 training begins when the optimal routine for the second task has yet to be established. Therefore, the established routine for the first task may influence the acquisition of the optimal routine for the second task. In the present study, the established routines for the backward circle span task might hinder the development of the optimal routines for the backward letter span task (Experiment 1), but not vice versa (Experiment 2). In other words, participants cannot generate optimal routines for the backward letter span task after the training of the backward circle span task, while the generation of optimal routines for the backward circle span task is unaffected by the existing routines acquired in the training on the backward letter span task. We speculate that the established routines for the backward circle span task might be carried over to the subsequent training phase, where they would no longer be optimal. This creates something similar to “functional fixation” or “inertia” that renders the acquisition process of the optimal backward letter routines less efficient or results in the development of suboptimal routines.

Although rarely mentioned in the field of WM training, phenomena similar to those found in the present study have been reported in the literature. For example, Poulton and Freeman (1966) summarized the unwanted asymmetric transfer effects in various task domains that could confound the experimental manipulations in studies using counterbalanced within-subject designs. For instance, in tasks involving the rehearsal of internal speech, performance in a quiet condition after a continuous noise condition may be worse than when the task is performed only in the quiet condition, probably due to the transfer of unsuitable strategies. By contrast, the performance in the continuous noise condition may be better with than without a transfer from the quiet condition (Aldridge, 1978; Poulton, 1979). However, despite their different task paradigms and domains, the phenomena in the above studies do share a common structure with those of the present study in terms of the negative transfer caused by the unsuitable use of previously acquired strategies and asymmetric pattern when the task order is reversed.

Specifically, regarding the asymmetry between the impacts of the backward circle training on the backward letter training and vice versa, we propose that its source may lie in the fundamental differences between the two task domains. Indeed, there is evidence of differences in the way verbal and spatial stimuli are handled in backward recall tasks. While the recall of verbal material is usually slower and less accurate backward than forward, the recall performance of spatial material is often equivalent (Donolato et al., 2017). Therefore, shifting between forward and backward recall is likely to involve different cognitive operations in the two domains. This suggests distinctive ways of representing sequences in these domains, corresponding to the distinctive functions of verbal and spatial WM (Gathercole et al., 2019; Norris et al., 2019b). Verbal WM favors the forward-going direction, as inputs such as words and sentences must be represented and processed in the original order. Hence, performing verbal backward recall is highly unfamiliar and challenging, and it requires distinctive routines to meet the specific requirements of the task. On the contrary, spatial WM may not inherently encode the input sequences in a forward fashion, as spatial representations are often needed when navigating using a sequence of spatial directions or backtracking to an earlier spatial location (Norris et al., 2019b). As a consequence, more inventive cognitive routines may be required to support backward recall training using verbal material. By contrast, backward recall training using spatial material does not necessitate elaborate routine development for the backward direction and even for spatial stimuli. Therefore, the spatial routine can also be used for verbal backward training but would be suboptimal for most individuals. The fact that these spatial reversal routines are already in place may nonetheless bias the form of the routine constructed in the backward letter training, leading to the training cost seen in Phase 2 of Experiment 1. On the contrary, any such routines developed for the previous verbal backward training would be verbal stimuli-specific and thus could not be applied to the later spatial backward training, as they would be irrelevant. Consequently, the development of optimal routines for later training remains unexplored, as we found no training cost in Experiment 2. After the consolidation of certain routines by intensive training, perhaps it is this difference in the two task routines, stemming from the fundamental differences in the representational mediums for the two domains, that give rise to the asymmetric negative transfer effect when shifting between verbal and spatial backward training.

### Supplementary information

ESM 1 (DOCX 93 kb)
