# Supplementary material for: Asymmetric negative transfer effects of working memory training
Source: Mem Cognit. 2023 Apr 21;51(7):1654–69. doi: 10.3758/s13421-023-01412-8 (PMC10520134; doi:10.3758/s13421-023-01412-8)
Supplement: Supplementary file 1 — (DOCX 93 kb) [file 13421_2023_1412_MOESM1_ESM.docx]

Asymmetric negative transfer effects of working memory training
Supplementary Materials

Nan Ni ^a^, Susan E. Gathercole ^b, c^, Dennis Norris ^c^, and Satoru Saito ^a^

^a^ Graduate School of Education, Kyoto University

^b^ Department of Psychiatry, University of Cambridge

^c^ MRC Cognition and Brain Sciences Unit, University of Cambridge

Author Note

This research was supported by JSPS KAKENHI Grant Number JP20K20861.

Corresponding authors: Graduate School of Education, Kyoto University, Yoshida-honmachi, Sakyo-ku, Kyoto, 606-8501, Japan (N. Ni). E-mail addresses: [nanni.kyt@gmail.com](mailto:nanni.kyt@gmail.com) (N. Ni) [saito.satoru.2z@kyoto-u.ac.jp](mailto:saito.satoru.2z@kyoto-u.ac.jp) (S. Saito).

Asymmetric negative transfer effects of working memory training
Supplementary Materials

# A. Pilot experiment of Experiment 1

## 1. Participants

Fifteen undergraduate and graduate Kyoto University students meeting the same criteria as in Experiment 1 were recruited and 5000 Japanese yen to participate. They were randomly allocated to three training conditions of Phase 1 (backward digit span, backward circle span, and color change detection), thus there were five participants in each training condition. Table S1 summarizes the demographic characteristics of the participants.

Table S1

*Demographics of Pilot Experiment.*

|  | Digit | Circle | Color |
| --- | --- | --- | --- |
| *N* | 5 | 5 | 5 |
| Age | 21.8 (2.05) | 21.6 (1.82) | 21.4 (1.14) |
| Gender F/M | 2/3 | 2/3 | 1/4 |

Note: Values in parentheses are standard deviations.

## 2. Procedure

The training design and training tasks of the pilot experiment were identical to Experiment 1. In Phase 1, the participants completed either the (1) backward digit span, (2) backward circle span, or (3) color change detection training for three sessions. In Phase 2, all participants completed the backward letter span training for three sessions. The participants received oral instruction about the experiment and completed the first session of the Phase 1 training in the laboratory environment. Then, the participants were instructed to complete the second and third sessions of the Phase 1 training using their personal computers online. After that, the participants returned to the laboratory and completed the first session of the Phase 2 training. In the end, the participants completed the second and third sessions of the Phase 2 training online.

## 3. Results

Figure S1 shows the highest scores achieved for each training session in the two phases. For Phase 1, repeated measures ANOVAs were performed on the scores in each session for each training group. There were significant increases on the performance across the training in each group: backward digit, *F*(2, 8) = 9.043, MSE = .383, *p* < .01, η_p_^2^ = 0.693; backward circle, *F*(2, 8) = 7.429, MSE = .117, *p* < .05, η_p_^2^ = 0.650; and color change detection, *F*(2, 8) = 42.957, MSE = .383, *p* < .001, η_p_^2^ = 0.915.

Figure S1. Performance changes in the pilot experiment. The left panel shows the performance changes of each training group in Phase 1. The right shows the performance changes of the backward letter training in Phase 2. The error bars represent 95% within-subject confidence intervals.

For Phase 2, a 3 (group) × 3 (training session) mixed ANOVA also established a significant main effect of the training session, *F*(2, 24) = 8.235, MSE = .472, *p* < .01, η_p_^2^ = 0.407. Importantly, there was a significant main effect of the Phase 1 training group, *F*(2, 12) = 10.684, MSE = 1.756, *p* < .01, η_p_^2^ = 0.640. The post hoc analyses of averaged performance using the Holm correction revealed that the circle group had significantly lower scores than the digit group and the color group (active control). The group × training session interaction was not significant (*F*(4, 24) = 0.471, MSE = .472, *p* = .757, η_p_^2^ = 0.073).

# B. Linear mixed-effects analysis results of Experiment 1 Phase 2

Formula: Span ~ 1 + LetterB_Span + Training_Group * P2_Block + (1 | ID)

“Span” is the span of each training block in Phase 2. “LetterB_Span” is the covariate, the span of the baseline backward letter task. “Training_Group” is the training condition in Phase 1 (backward digit span, backward circle span, or color change detection). “P2_Block” is the number of the training block in Phase 2.

Table S2

*Experiment 1 Phase 2 ANOVA Summary*

| Effect | df | F | p |
| --- | --- | --- | --- |
| LetterB_Span | 1, 41.00 | 99.023 | < .001 |
| Training_Group | 2, 41.00 | 13.021 | < .001 |
| P2_Block | 23, 966.00 | 90.600 | < .001 |
| Training_Group * P2_Block | 46, 966.00 | 1.865 | < .001 |

Note: Model terms tested with Satterthwaite method.

Table S3

*Experiment 1 Phase 2 Fixed Effects Estimates*

| Term | Estimate | SE | df | t | p |
| --- | --- | --- | --- | --- | --- |
| Intercept | 3.056 | 0.326 | 41.000 | 9.365 | < .001 |
| LetterB_Span | 0.634 | 0.064 | 41.000 | 9.951 | < .001 |
| Training_Group (1) | 0.558 | 0.118 | 41.000 | 4.714 | < .001 |
| Training_Group (2) | -0.498 | 0.121 | 41.000 | -4.131 | < .001 |
| P2_Block (1) | -3.195 | 0.104 | 966.000 | -30.864 | < .001 |
| P2_Block (2) | -2.352 | 0.104 | 966.000 | -22.726 | < .001 |
| P2_Block (3) | -1.465 | 0.104 | 966.000 | -14.157 | < .001 |
| P2_Block (4) | -0.755 | 0.104 | 966.000 | -7.299 | < .001 |
| P2_Block (5) | -0.343 | 0.104 | 966.000 | -3.312 | < .001 |
| P2_Block (6) | -0.103 | 0.104 | 966.000 | -0.998 | 0.319 |
| P2_Block (7) | -0.032 | 0.104 | 966.000 | -0.308 | 0.758 |
| P2_Block (8) | -0.008 | 0.104 | 966.000 | -0.078 | 0.938 |
| P2_Block (9) | 0.127 | 0.104 | 966.000 | 1.228 | 0.220 |
| P2_Block (10) | 0.435 | 0.104 | 966.000 | 4.199 | < .001 |
| P2_Block (11) | 0.414 | 0.104 | 966.000 | 3.995 | < .001 |
| P2_Block (12) | 0.305 | 0.104 | 966.000 | 2.947 | 0.003 |
| P2_Block (13) | 0.433 | 0.104 | 966.000 | 4.181 | < .001 |
| P2_Block (14) | 0.485 | 0.104 | 966.000 | 4.682 | < .001 |
| P2_Block (15) | 0.458 | 0.104 | 966.000 | 4.423 | < .001 |
| P2_Block (16) | 0.463 | 0.104 | 966.000 | 4.469 | < .001 |
| P2_Block (17) | 0.354 | 0.104 | 966.000 | 3.420 | < .001 |
| P2_Block (18) | 0.676 | 0.104 | 966.000 | 6.533 | < .001 |
| P2_Block (19) | 0.648 | 0.104 | 966.000 | 6.259 | < .001 |
| P2_Block (20) | 0.694 | 0.104 | 966.000 | 6.706 | < .001 |
| P2_Block (21) | 0.699 | 0.104 | 966.000 | 6.750 | < .001 |
| P2_Block (22) | 0.699 | 0.104 | 966.000 | 6.750 | < .001 |
| P2_Block (23) | 0.706 | 0.104 | 966.000 | 6.823 | < .001 |
| Training_Group (1) * P2 _Block (1) | -0.547 | 0.146 | 966.000 | -3.739 | < .001 |
| Training_Group (2) * P2 _Block (1) | 0.558 | 0.149 | 966.000 | 3.748 | < .001 |
| Training_Group (1) * P2_Block (2) | -0.523 | 0.146 | 966.000 | -3.573 | < .001 |
| Training_Group (2) * P2_Block (2) | 0.501 | 0.149 | 966.000 | 3.366 | < .001 |
| Training_Group (1) * P2_Block (3) | -0.410 | 0.146 | 966.000 | -2.800 | 0.005 |
| Training_Group (2) * P2_Block (3) | 0.400 | 0.149 | 966.000 | 2.687 | 0.007 |
| Training_Group (1) * P2_Block (4) | -0.186 | 0.146 | 966.000 | -1.273 | 0.203 |
| Training_Group (2) * P2_Block (4) | 0.261 | 0.149 | 966.000 | 1.756 | 0.079 |
| Training_Group (1) * P2_Block (5) | 0.068 | 0.146 | 966.000 | 0.463 | 0.643 |
| Training_Group (2) * P2_Block (5) | -0.080 | 0.149 | 966.000 | -0.536 | 0.592 |
| Training_Group (1) * P2_Block (6) | -0.105 | 0.146 | 966.000 | -0.718 | 0.473 |
| Training_Group (2) * P2_Block (6) | -0.105 | 0.149 | 966.000 | -0.706 | 0.481 |
| Training_Group (1) * P2_Block (7) | -0.176 | 0.146 | 966.000 | -1.206 | 0.228 |
| Training_Group (2) * P2_Block (7) | 0.038 | 0.149 | 966.000 | 0.254 | 0.799 |
| Training_Group (1) * P2_Block (8) | -0.200 | 0.146 | 966.000 | -1.369 | 0.171 |
| Training_Group (2) * P2_Block (8) | 0.085 | 0.149 | 966.000 | 0.574 | 0.566 |
| Training_Group (1) * P2_Block (9) | -0.135 | 0.146 | 966.000 | -0.926 | 0.355 |
| Training_Group (2) * P2_Block (9) | 0.093 | 0.149 | 966.000 | 0.626 | 0.531 |
| Training_Group (1) * P2_Block (10) | -0.110 | 0.146 | 966.000 | -0.749 | 0.454 |
| Training_Group (2) * P2_Block (10) | -7.440e^-5^ | 0.149 | 966.000 | -4.998e^-4^ | 1.000 |
| Training_Group (1) * P2_Block (11) | 0.045 | 0.146 | 966.000 | 0.306 | 0.760 |
| Training_Group (2) * P2_Block (11) | -0.050 | 0.149 | 966.000 | -0.339 | 0.735 |
| Training_Group (1) * P2_Block (12) | 0.087 | 0.146 | 966.000 | 0.592 | 0.554 |
| Training_Group (2) * P2_Block (12) | -0.013 | 0.149 | 966.000 | -0.090 | 0.928 |
| Training_Group (1) * P2_Block (13) | 0.092 | 0.146 | 966.000 | 0.630 | 0.529 |
| Training_Group (2) * P2_Block (13) | -0.141 | 0.149 | 966.000 | -0.948 | 0.343 |
| Training_Group (1) * P2_Block (14) | 0.240 | 0.146 | 966.000 | 1.643 | 0.101 |
| Training_Group (2) * P2_Block (14) | -0.050 | 0.149 | 966.000 | -0.336 | 0.737 |
| Training_Group (1) * P2_Block (15) | 0.267 | 0.146 | 966.000 | 1.827 | 0.068 |
| Training_Group (2) * P2_Block (15) | -0.166 | 0.149 | 966.000 | -1.116 | 0.265 |
| Training_Group (1) * P2_Block (16) | 0.062 | 0.146 | 966.000 | 0.427 | 0.670 |
| Training_Group (2) * P2_Block (16) | 0.043 | 0.149 | 966.000 | 0.291 | 0.771 |
| Training_Group (1) * P2_Block (17) | 0.104 | 0.146 | 966.000 | 0.713 | 0.476 |
| Training_Group (2) * P2_Block (17) | 0.080 | 0.149 | 966.000 | 0.541 | 0.589 |
| Training_Group (1) * P2_Block (18) | 0.249 | 0.146 | 966.000 | 1.700 | 0.089 |
| Training_Group (2) * P2_Block (18) | -0.242 | 0.149 | 966.000 | -1.624 | 0.105 |
| Training_Group (1) * P2_Block (19) | 0.344 | 0.146 | 966.000 | 2.350 | 0.019 |
| Training_Group (2) * P2_Block (19) | -0.428 | 0.149 | 966.000 | -2.873 | 0.004 |
| Training_Group (1) * P2_Block (20) | 0.231 | 0.146 | 966.000 | 1.578 | 0.115 |
| Training_Group (2) * P2_Block (20) | -0.331 | 0.149 | 966.000 | -2.224 | 0.026 |
| Training_Group (1) * P2_Block (21) | 0.160 | 0.146 | 966.000 | 1.091 | 0.275 |
| Training_Group (2) * P2_Block (21) | -0.193 | 0.149 | 966.000 | -1.295 | 0.196 |
| Training_Group (1) * P2_Block (22) | 0.160 | 0.146 | 966.000 | 1.091 | 0.275 |
| Training_Group (2) * P2_Block (22) | -0.193 | 0.149 | 966.000 | -1.295 | 0.196 |
| Training_Group (1) * P2_Block (23) | 0.085 | 0.146 | 966.000 | 0.584 | 0.559 |
| Training_Group (2) * P2_Block (23) | 0.014 | 0.149 | 966.000 | 0.094 | 0.925 |

Note: The intercept corresponds to the (unweighted) grand mean; for each factor with k levels, k - 1 parameters are estimated. Consequently, the estimates cannot be directly mapped to factor levels.

# C. Linear mixed-effects analysis results of Experiment 2 Phase 2

Formula: Span ~ 1 + CircleB_Span + Training_Group * P2_Training_Block + (1 | ID)

“Span” is the span of each training block in Phase 2. “CircleB_Span” is the covariate, the span of the baseline backward circle task. “Training_Group” is the training condition in Phase 1 (backward square span, backward letter span, or color change detection). “P2_Block” is the number of the training block in Phase 2.

Table S4

*Experiment 2 Phase 2 ANOVA Summary*

| Effect | df | F | p |
| --- | --- | --- | --- |
| CircleB_Span | 1, 43.00 | 9.589 | 0.003 |
| Training_Group | 2, 43.00 | 4.306 | 0.020 |
| P2_Block | 23, 1012.00 | 183.965 | < .001 |
| Training_Group * P2_Block | 46, 1012 | 2.246 | < .001 |

Note: Model terms tested with Satterthwaite method.

Table S5

*Experiment 2 Phase 2 Fixed Effects Estimates*

| Term | Estimate | SE | df | t | p |
| --- | --- | --- | --- | --- | --- |
| Intercept | 4.520 | 0.755 | 43.000 | 5.983 | < .001 |
| CircleB_Span | 0.397 | 0.128 | 43.000 | 3.097 | 0.003 |
| Training_Group (1) | 0.403 | 0.148 | 43.000 | 2.716 | 0.009 |
| Training_Group (2) | -0.039 | 0.150 | 43.000 | -0.262 | 0.794 |
| P2_Block (1) | -3.835 | 0.088 | 1012.000 | -43.574 | < .001 |
| P2_Block (2) | -2.856 | 0.088 | 1012.000 | -32.449 | < .001 |
| P2_Block (3) | -1.876 | 0.088 | 1012.000 | -21.310 | < .001 |
| P2_Block (4) | -1.070 | 0.088 | 1012.000 | -12.152 | < .001 |
| P2_Block (5) | -0.527 | 0.088 | 1012.000 | -5.989 | < .001 |
| P2_Block (6) | -0.048 | 0.088 | 1012.000 | -0.543 | 0.587 |
| P2_Block (7) | 0.181 | 0.088 | 1012.000 | 2.059 | 0.040 |
| P2_Block (8) | 0.238 | 0.088 | 1012.000 | 2.701 | 0.007 |
| P2_Block (9) | 0.280 | 0.088 | 1012.000 | 3.180 | 0.002 |
| P2_Block (10) | 0.453 | 0.088 | 1012.000 | 5.148 | < .001 |
| P2_Block (11) | 0.531 | 0.088 | 1012.000 | 6.033 | < .001 |
| P2_Block (12) | 0.488 | 0.088 | 1012.000 | 5.540 | < .001 |
| P2_Block (13) | 0.555 | 0.088 | 1012.000 | 6.303 | < .001 |
| P2_Block (14) | 0.574 | 0.088 | 1012.000 | 6.520 | < .001 |
| P2_Block (15) | 0.556 | 0.088 | 1012.000 | 6.317 | < .001 |
| P2_Block (16) | 0.535 | 0.088 | 1012.000 | 6.081 | < .001 |
| P2_Block (17) | 0.559 | 0.088 | 1012.000 | 6.351 | < .001 |
| P2_Block (18) | 0.749 | 0.088 | 1012.000 | 8.507 | < .001 |
| P2_Block (19) | 0.782 | 0.088 | 1012.000 | 8.885 | < .001 |
| P2_Block (20) | 0.768 | 0.088 | 1012.000 | 8.730 | < .001 |
| P2_Block (21) | 0.757 | 0.088 | 1012.000 | 8.603 | < .001 |
| P2_Block (22) | 0.733 | 0.088 | 1012.000 | 8.332 | < .001 |
| P2_Block (23) | 0.718 | 0.088 | 1012.000 | 8.157 | < .001 |
| Training_Group (1) * P2 _Block (1) | -0.449 | 0.122 | 1012.000 | -3.688 | < .001 |
| Training_Group (2) * P2 _Block (1) | 0.069 | 0.124 | 1012.000 | 0.562 | 0.574 |
| Training_Group (1) * P2_Block (2) | -0.428 | 0.122 | 1012.000 | -3.517 | < .001 |
| Training_Group (2) * P2_Block (2) | 0.028 | 0.124 | 1012.000 | 0.225 | 0.822 |
| Training_Group (1) * P2_Block (3) | -0.468 | 0.122 | 1012.000 | -3.839 | < .001 |
| Training_Group (2) * P2_Block (3) | 0.047 | 0.124 | 1012.000 | 0.384 | 0.701 |
| Training_Group (1) * P2_Block (4) | -0.391 | 0.122 | 1012.000 | -3.212 | 0.001 |
| Training_Group (2) * P2_Block (4) | -0.009 | 0.124 | 1012.000 | -0.070 | 0.945 |
| Training_Group (1) * P2_Block (5) | -0.110 | 0.122 | 1012.000 | -0.904 | 0.366 |
| Training_Group (2) * P2_Block (5) | -0.176 | 0.124 | 1012.000 | -1.424 | 0.155 |
| Training_Group (1) * P2_Block (6) | 0.116 | 0.122 | 1012.000 | 0.956 | 0.339 |
| Training_Group (2) * P2_Block (6) | -0.280 | 0.124 | 1012.000 | -2.268 | 0.024 |
| Training_Group (1) * P2_Block (7) | 0.182 | 0.122 | 1012.000 | 1.490 | 0.136 |
| Training_Group (2) * P2_Block (7) | -0.259 | 0.124 | 1012.000 | -2.098 | 0.036 |
| Training_Group (1) * P2_Block (8) | 0.125 | 0.122 | 1012.000 | 1.026 | 0.305 |
| Training_Group (2) * P2_Block (8) | -0.003 | 0.124 | 1012.000 | -0.027 | 0.978 |
| Training_Group (1) * P2_Block (9) | 0.200 | 0.122 | 1012.000 | 1.646 | 0.100 |
| Training_Group (2) * P2_Block (9) | -0.108 | 0.124 | 1012.000 | -0.874 | 0.382 |
| Training_Group (1) * P2_Block (10) | 0.145 | 0.122 | 1012.000 | 1.190 | 0.234 |
| Training_Group (2) * P2_Block (10) | -0.094 | 0.124 | 1012.000 | -0.758 | 0.449 |
| Training_Group (1) * P2_Block (11) | 0.185 | 0.122 | 1012.000 | 1.516 | 0.130 |
| Training_Group (2) * P2_Block (11) | 0.016 | 0.124 | 1012.000 | 0.129 | 0.898 |
| Training_Group (1) * P2_Block (12) | 0.169 | 0.122 | 1012.000 | 1.390 | 0.165 |
| Training_Group (2) * P2_Block (12) | 0.059 | 0.124 | 1012.000 | 0.480 | 0.631 |
| Training_Group (1) * P2_Block (13) | 0.161 | 0.122 | 1012.000 | 1.321 | 0.187 |
| Training_Group (2) * P2_Block (13) | -0.008 | 0.124 | 1012.000 | -0.064 | 0.949 |
| Training_Group (1) * P2_Block (14) | 0.083 | 0.122 | 1012.000 | 0.681 | 0.496 |
| Training_Group (2) * P2_Block (14) | 0.161 | 0.124 | 1012.000 | 1.298 | 0.194 |
| Training_Group (1) * P2_Block (15) | 0.101 | 0.122 | 1012.000 | 0.828 | 0.408 |
| Training_Group (2) * P2_Block (15) | 0.053 | 0.124 | 1012.000 | 0.432 | 0.666 |
| Training_Group (1) * P2_Block (16) | 0.122 | 0.122 | 1012.000 | 0.999 | 0.318 |
| Training_Group (2) * P2_Block (16) | 0.012 | 0.124 | 1012.000 | 0.095 | 0.925 |
| Training_Group (1) * P2_Block (17) | 0.098 | 0.122 | 1012.000 | 0.804 | 0.422 |
| Training_Group (2) * P2_Block (17) | -0.012 | 0.124 | 1012.000 | -0.098 | 0.922 |
| Training_Group (1) * P2_Block (18) | 0.085 | 0.122 | 1012.000 | 0.694 | 0.488 |
| Training_Group (2) * P2_Block (18) | 0.048 | 0.124 | 1012.000 | 0.389 | 0.697 |
| Training_Group (1) * P2_Block (19) | 0.169 | 0.122 | 1012.000 | 1.387 | 0.166 |
| Training_Group (2) * P2_Block (19) | 0.140 | 0.124 | 1012.000 | 1.131 | 0.258 |
| Training_Group (1) * P2_Block (20) | 0.124 | 0.122 | 1012.000 | 1.016 | 0.310 |
| Training_Group (2) * P2_Block (20) | 0.029 | 0.124 | 1012.000 | 0.231 | 0.818 |
| Training_Group (1) * P2_Block (21) | -0.041 | 0.122 | 1012.000 | -0.340 | 0.734 |
| Training_Group (2) * P2_Block (21) | 0.040 | 0.124 | 1012.000 | 0.321 | 0.748 |
| Training_Group (1) * P2_Block (22) | -0.018 | 0.122 | 1012.000 | -0.145 | 0.885 |
| Training_Group (2) * P2_Block (22) | 0.064 | 0.124 | 1012.000 | 0.514 | 0.607 |
| Training_Group (1) * P2_Block (23) | -0.120 | 0.122 | 1012.000 | -0.984 | 0.325 |
| Training_Group (2) * P2_Block (23) | 0.079 | 0.124 | 1012.000 | 0.638 | 0.523 |

Note: The intercept corresponds to the (unweighted) grand mean; for each factor with k levels, k - 1 parameters are estimated. Consequently, the estimates cannot be directly mapped to factor levels.

# D. Strategy questionnaire

Table S6

*Descriptions of the Strategy Questions*

| Strategy No. | Description |
| --- | --- |
| 1 | Rehearse the items as they were presented. |
| 2 | Group the items by separating them into sets of particular sizes. |
| 3 | Group the items according to the pattern they form. |
| 4 | Group the items according to their meaning. |
| 5 | Form a mental image. |
| 6 | Hold the items in mind and try to recall them backwards from the last one first. |
| 7 | Run through the list forwards to the last item, and then repeat this for the item before the last one, and so on. |
| 8 | Reverse the order of items (for example, in pairs or more of the items) as they are being presented. |
| 9 | Reverse the order of items (for example, in pairs) just before they are recalled. |
| 10 | Remember the last few items first and then do something else for the early items in the list. |
| 11 | Use the panel as recall cues. (Remember items as they were presented. When recalling, recall items in the exact order and remember the position of buttons. Then click buttons in the reversed order just like in backward circle span task.) |

# E. Strategy questionnaire analyses results of Experiment 1 Phase 2

Table S7

*Strategy Use Results of Experiment 1 Phase 2 Backward Circle Training*

| Strategy No. | Digit group | Circle group | Color group | *p* | η_p_^2^ | BF_10_ |
| --- | --- | --- | --- | --- | --- | --- |
| 1 | 2.267 (1.163) | 2.357 (0.745) | 2.250 (1.125) | 0.956 | 0.002 | 0.170 |
| 2 | 2.533 (0.915) | 1.857 (1.231) | 2.500 (0.894) | 0.144 | 0.088 | 0.659 |
| 3 | 1.067 (1.100) | 1.000 (1.109) | 0.625 (0.885) | 0.444 | 0.038 | 0.294 |
| 4 | 1.133 (0.915) | 0.714 (0.914) | 1.000 (0.894) | 0.455 | 0.037 | 0.286 |
| 5 | 0.800 (1.082) | 0.857 (0.949) | 0.875 (0.885) | 0.976 | 0.001 | 0.167 |
| 6 | 1.467 (1.246) | 1.500 (1.160) | 1.438 (1.365) | 0.991 | 4.340e^-4^ | 0.166 |
| 7 | 0.667 (0.976) | 0.929 (0.917) | 1.125 (1.258) | 0.496 | 0.033 | 0.272 |
| 8 | 0.467 (1.060) | 0.214 (0.802) | 0 (0) | 0.241 | 0.065 | 0.460 |
| 9 | 1.267 (1.223) | 1.357 (1.082) | 2.063 (1.340) | 0.153 | 0.085 | 0.645 |
| 10 | 1.800 (1.082) | 1.429 (1.089) | 1.875 (0.806) | 0.438 | 0.039 | 0.294 |
| 11 | 0.733 (1.033) | 0.786 (1.051) | 0.813 (1.328) | 0.981 | 8.929e^-4^ | 0.167 |

Note: Values in parentheses are standard deviations.

# F. Strategy questionnaire analyses results of Experiment 2 Phase 2

Table S8

*Strategy Use Results of Experiment 2 Phase 2 Backward Letter Training*

| Strategy No. | Square group | Letter group | Color group | *p* | η_p_^2^ | BF_10_ |
| --- | --- | --- | --- | --- | --- | --- |
| 1 | 1.529 (1.281) | 1.938 (1.124) | 0.857 (0.864) | < .05 | 0.138 | 1.773 |
| 2 | 2.412 (0.870) | 2.125 (1.204) | 1.500 (1.160) | 0.071 | 0.113 | 1.098 |
| 3 | 2.000 (1.000) | 1.688 (1.014) | 0.857 (0.864) | < .01 | 0.203 | 6.884 |
| 4 | 0.941 (1.088) | 0.313 (0.602) | 0.357 (0.633) | 0.061 | 0.120 | 1.296 |
| 5 | 2.000 (0.935) | 1.750 (1.000) | 1.714 (1.069) | 0.678 | 0.017 | 0.211 |
| 6 | 1.412 (1.004) | 2.000 (1.155) | 1.286 (1.326) | 0.197 | 0.071 | 0.518 |
| 7 | 0.353 (0.702) | 0.563 (0.892) | 0.214 (0.579) | 0.436 | 0.037 | 0.288 |
| 8 | 0.294 (0.772) | 0.188 (0.403) | 0.357 (0.633) | 0.753 | 0.013 | 0.195 |
| 9 | 1.882 (1.166) | 2.000 (1.095) | 1.143 (0.949) | 0.077 | 0.110 | 1.022 |
| 10 | 1.941 (1.029) | 1.750 (1.125) | 1.857 (1.099) | 0.880 | 0.006 | 0.175 |

Note: Values in parentheses are standard deviations.
